# Identification of a Novel Deltavirus in Boa Constrictors

**DOI:** 10.1128/mBio.00014-19

**Published:** 2019-04-02

**Authors:** Udo Hetzel, Leonóra Szirovicza, Teemu Smura, Barbara Prähauser, Olli Vapalahti, Anja Kipar, Jussi Hepojoki

**Affiliations:** a Institute of Veterinary Pathology, Vetsuisse Faculty, University of Zurich, Zurich, Switzerland; b Department of Veterinary Biosciences, Faculty of Veterinary Medicine, University of Helsinki, Helsinki, Finland; c Department of Virology, Faculty of Medicine, Medicum, University of Helsinki, Helsinki, Finland; d Department of Virology and Immunology, HUSLAB, Helsinki University Hospital, Helsinki, Finland; Columbia University Medical College

**Keywords:** deltavirus, evolutionary biology, hepatitis, virology, zoonotic infections

## Abstract

Hepatitis D virus (HDV) forms the genus *Deltavirus* unassigned to any virus family. HDV is a satellite virus and needs hepatitis B virus (HBV) to make infectious particles. Deltaviruses are thought to have evolved in humans, since for a long time, they had not been identified elsewhere. Herein we report, prompted by the recent discovery of an HDV-like agent in birds, the identification of a deltavirus in snakes (Boa constrictor) designated snake HDV (sHDV). The circular 1,711-nt RNA genome of sHDV resembles human HDV (hHDV) in its coding strategy and size. We discovered sHDV during a metatranscriptomic study of brain samples of a *Boa constrictor* breeding pair with central nervous system signs. Applying next-generation sequencing (NGS) to brain, blood, and liver samples from both snakes, we did not find reads matching hepadnaviruses. Sequence comparison showed the snake delta antigen (sHDAg) to be 55% and 37% identical to its human and avian counterparts. Antiserum raised against recombinant sHDAg was used in immunohistology and demonstrated a broad viral target cell spectrum, including neurons, epithelial cells, and leukocytes. Using RT-PCR, we also detected sHDV RNA in two juvenile offspring and in a water python (Liasis mackloti *savuensis*) in the same snake colony, potentially indicating vertical and horizontal transmission. Screening of 20 randomly selected boas from another breeder by RT-PCR revealed sHDV infection in three additional snakes. The observed broad tissue tropism and the failure to detect accompanying hepadnavirus suggest that sHDV could be a satellite virus of a currently unknown enveloped virus.

## Abstract (precis)

So far, the only known example of deltaviruses is the hepatitis delta virus (HDV). HDV is speculated to have evolved in humans, since deltaviruses were until very recently found only in humans. Using a metatranscriptomic sequencing approach, we found a circular RNA, which resembles that of HDV in size and coding strategy, in a snake. The identification of similar deltaviruses in distantly related species other than humans indicates that the previously suggested hypotheses on the origins of deltaviruses need to be updated. It is still possible that the ancestor of deltaviruses emerged from cellular RNAs; however, it likely would have happened much earlier in evolution than previously thought. These findings open up completely new avenues in evolution and pathogenesis studies of deltaviruses.

## OBSERVATION

Hepatitis D virus (HDV) forms and is the sole member of the genus *Deltavirus* so far (1). Until the very recent finding of HDV-like sequences in ducks (2), HDV had been found only in humans, and it is represented by eight distinct genotypes (1, 3). In fact, HDV is hypothesized to have evolved within the human host (4). HDV has a negative-sense single-stranded circular RNA genome of 1,672 to 1,697 nucleotides which is highly self-complementary (3, 5). Processing (autocatalytic cleavage of multimeric genomic and antigenomic RNAs and ligation of monomers) of the genome is mediated by genomic and antigenomic ribozymes (3, 6). HDV encodes only two proteins, the small and large hepatitis delta antigens (S- and L-HDAg), which are identical in amino acid sequence except that the L-HDAg contains 19 additional amino acid residues at its C terminus (7). The S-HDAg is needed for RNA replication, and the L-HDAg is involved in virus assembly (7). The virus requires hepatitis B virus (HBV) for egress and formation of infectious particles comprising a ribonucleoprotein formed of the circular RNA genome and HDAgs within an envelope decorated with HBV S antigen (3, 8). HDV replicates in the nucleus, and the evidence suggests that cellular DNA-dependent RNA polymerase II mediates HDV RNA replication (7). Patients with chronic HBV and HDV coinfection are at great risk of developing liver cirrhosis and hepatocellular carcinoma, particularly in the case of superinfection with HDV in a chronically HBV-infected patient (3). The HDV prevalence among HBV carriers is estimated to be around 5% (3); however, it varies greatly depending on geographical area and viral genotype (9). Very recently, sequence data showing the presence of a divergent HDV-like agent was reported in ducks, without any traces of duck orthohepadnavirus (2). This prompted us to report our findings of an HDV-like agent which we discovered in snakes in early 2018.

The animals included in this study were boa constrictor snakes submitted to the Institute of Veterinary Pathology, Vetsuisse Faculty, University of Zurich, Switzerland for euthanasia due to suspected boid inclusion body disease (BIBD) and subsequent diagnostic postmortem examination upon the owner’s request. We applied the Animals Scientific Procedures Act 1986 (ASPA), schedule 1 (http://www.legislation.gov.uk/ukpga/1986/14/schedule/1) procedure to euthanize the snakes. Euthanasia and diagnosis-motivated necropsies are both routine veterinary procedures, and thus, ethical permissions were not required. The blood samples used in the study were collected for diagnostic purposes.

**FIG 1:**
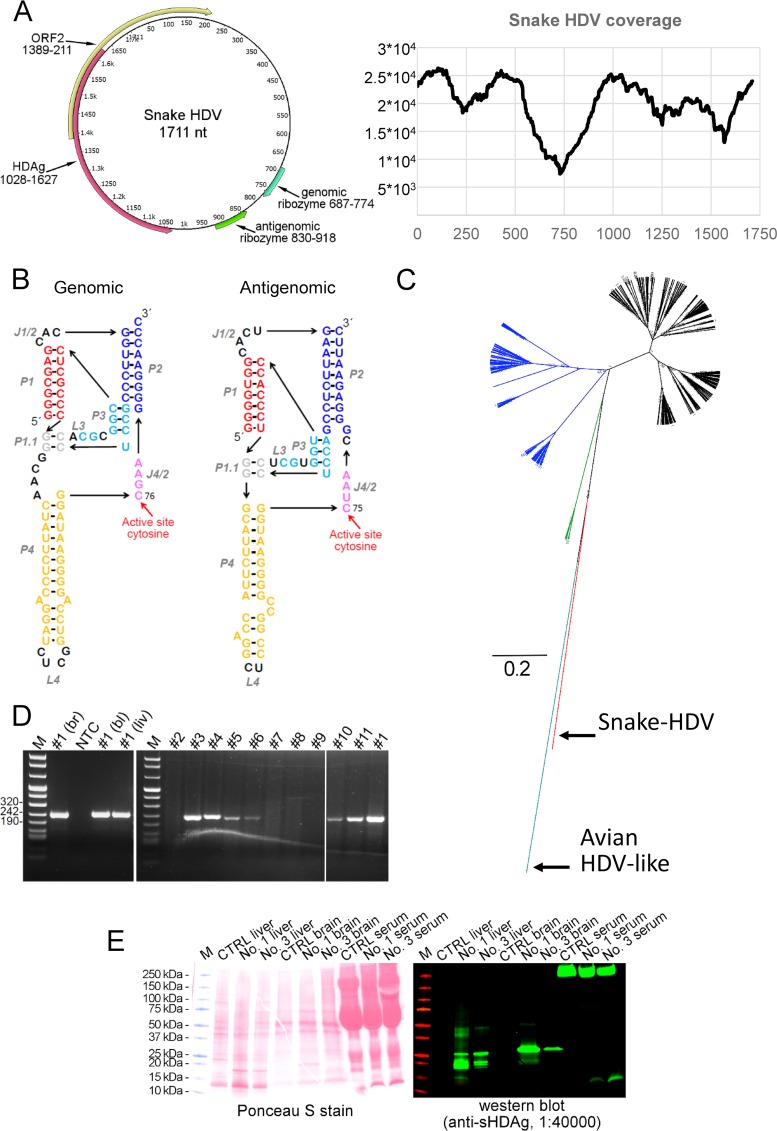
Genome organization, sequencing coverage, schematic ribozyme structure, and phylogenetic analysis of snake HDV. (A) Schematic presentation of circular RNA genome and sequencing coverage for snake HDV. The genome shows two open reading frames (ORFs). The ORF in the antigenomic orientation spanning nucleotide residues 1028 to 1627 encodes a 199-amino-acid protein which by BLAST analysis represents the HDAg. ORF2 is in the genomic orientation and spans residues 1389 to 211 and encodes a 177-amino-acid protein, which by BLAST analysis did not yield significant hits (35% identity over 66 amino acids (E value of 5) to ferritin-like protein from “*Candidatus* Nitrososphaera evergladensis” SR1, NCBI protein accession no. AIF82718.1). SMART (Simple Modular Architecture Research Tool available at http://smart.embl-heidelberg.de/) analysis showed the putative protein to have two transmembrane helices, and a DUF3343 (domain of unknown function) domain with an E value of 0.013. The genomic and antigenomic ribozymes identified by sequence alignments to known HDVs are located at nt 687 to 744 and 830 to 918, respectively. The graph shows sequencing coverage (on the *y* axis) in respect to each nucleotide position (on the *x* axis) of snake HDV from the original brain sample, and coverage ranges from 7,368 (at nt position 729) to 26,304-fold. (B) Models for the secondary structures of the genomic and antigenomic ribozymes identified in snake HDV. The presentation format is adopted from a review by Webb and Luptak (20) which was also used by Wille et al. (2). Paired regions (P), joining regions (J), and loops (L) are shown. Both genomic and antigenomic ribozymes are structurally close to their human HDV counterparts described in reference 20, and they are identical at the following regions: active site, P1.1, and P3. Cleavage by the ribozyme occurs at the 5′ end. (C) Phylogenetic analysis of human, avian, and reptile HDAgs. The phylogenetic analysis was done using Bayesian MCMC method implemented in MrBayes 3.1.2 (21) with the JTT model of substitution with gamma distributed rate variation among sites. HDV genotype 1 (black), HDV genotype 2 (blue), HDV genotype 3 (green), avian HDV-like sequence (cyan), and snake HDV (red) are indicated. (D) RT-PCR results of snake tissues. The gel on the left shows RT-PCR products obtained for snake 1 (Fig. 2H) from different tissues: brain (br), blood (bl), and liver (liv). NTC, nontemplate control, M is DNA ladder. The gel on the right shows RT-PCR products obtained from liver samples, the animal numbering is according to Fig. 2H, and animal 1 serves as a positive control. (E) Western blot of liver and brain homogenates and serum from sHDV RT-PCR negative-control animal (animal 7 [Fig. 2H]) and sHDV RT-PCR-positive animals (animals 1 and 3 [Fig. 2H]). The panel on the left shows total protein staining by Ponceau S, and the panel on the right shows staining with anti-sHDAg (1:40,000) antiserum using IRDye 800CW-conjugated donkey anti-rabbit IgG (LI-COR Biosciences). The signal for Western blot was read with Odyssey Infrared Imaging System (LI-COR Biosciences).

The animals carrying the snake HDV (sHDV) were a *Boa constrictor sabogae* breeding pair with their joint offspring (F2 and F3) and a water python (*Liasis mackloti savuensis*) from the same colony. The parental animals (animals 1 and 3) had originally been imported from Panama to Italy, from where they were sold to a private owner in Switzerland. All snakes had shown mild neurological signs, which were suspected to be associated with BIBD. Confirmation of BIBD was achieved by examination of blood smears. After euthanasia, the diagnosis was confirmed in both snakes by histological examination of formalin-fixed paraffin-embedded samples of brain and other tissues. Apart from the water python, which suffered from chronic hepatitis, none of the snakes exhibited other histopathological changes. We prepared next-generation sequencing (NGS) libraries using RNA extracted from the brain, liver, and blood of the parental animals (animals 1 and 3), both as described previously (10). The sequencing by the Illumina MiSeq platform with MiSeq Reagent kit v3 (Illumina) 2 × 300 cycles yielded 825,933 paired end reads for the brain sample of the father (animal 1), and removal of reads matching the snake genome (Python bivitattus) reduced the number of paired end reads to 401,141. We performed *de novo* assembly using MIRA version 4.9.5 (http://mira-assembler.sourceforge.net/) on CSC (IT Center for Science Ltd., Finland) Taito supercluster. One of the contigs with high coverage (130,902 reads in total, corresponding to 7.92% of all reads) appeared to be circular. The contig contained three repeats of a 1,711-nt sequence (GenBank accession no. MH988742) with two open reading frames (ORFs), one in the sense orientation and the other in the antisense orientation. The genome of the newly identified sHDV and the sequencing coverage are shown in Fig. 1A. High numbers of reads matching the same virus were found in the NGS libraries prepared from the brain, blood, and liver from both animals (animals 1 and 3). To look for accompanying hepadnaviruses, we collected 50 to 100 contigs with the highest coverage (reptarenaviruses, hartmaniviruses, snake genome, and bacterial sequences) for each sample, used them as bait to remove the uninteresting reads, and performed *de novo* assembly using MIRA version 4.9.5. After five cleaning rounds, we had approximately 40,000 contigs, which we analyzed using BLASTX (on CSC Taito supercluster) against hepadnavirus sequences in nr database, the contigs with matches (approximately 250) were reanalyzed using BLASTX against the entire nr database, and after removal of uninteresting contigs (snake and bacterial genomes), we analyzed the remaining reads using nucleotide BLAST at the NCBI website. With this approach, we could not detect hepadnavirus-like reads/contigs in any of the samples. However, based on the results, we cannot rule out the presence of an accompanying, previously unknown, hepadnavirus.

By BLAST analysis (https://blast.ncbi.nlm.nih.gov/Blast.cgi), the other ORF of 199 amino acids was identified as snake HDAg (sHDAg) with 55% amino acid identity to small hHDAg (S-hHDAg, NCBI protein accession no. AWI66689.1) and 37% to avian HDAg (AYC81245.1); sequence alignment and identities are presented in Fig. S1 in the supplemental material. The large hHDAg (L-hHDAg) is produced by dsRNA-adenosine deaminase-mediated editing of the UAG stop codon to yield UGG (tryptophan), thus yielding 19 additional amino acid residues (11). Also, the sHDAg ORF terminates with UAG, and similar editing would yield L-sHDAG with 22 additional residues. The putative L-sHDAg sequence is included in Fig. S1. The hHDAg locates almost exclusively to the nucleus, and by prediction (ELM [12] and NLStradamus [13]) also, sHDAg harbors several nuclear localization signals (NLSs) (Fig. S1). ORF2 contains a stretch resembling the DUF3343 (domain of unknown function) by HMMER3 search in SMART (Simple Modular Architecture Research Tool available at http://smart.embl-heidelberg.de/), but with no apparent other homologies to known proteins. The secondary structure of the genome generated using the RNAstructure webserver (14) shows 73% self-complementarity, which is close to the 74% reported for known hHDVs (3). By GC content (53.3%), the sHDV lies between the newly reported avian HDV-like sequence (51%) (2) and human HDV (hHDV) (60%) (5).

10.1128/mBio.00014-19.1 FIG S1 Amino acid identities between snake, human, and avian HDAgs. Amino acid alignment of the HDAg is shown at the top. Identical residues are indicated by dark gray shading. The table below the amino acid alignment shows the amino acid identities between the S-HDAgs. Below the table, the amino acid sequence of a putative large sHDAg is shown. The italic residues represent the NLS (nuclear localization signal) by Viterbi prediction in NLStradamus (13). The purple residues represent NLSs as predicted by ELM (eukaryotic linear motif search [12]). The underlined residues together with the blue residues represent NLSs by posterior prediction in NLStradamus (13). The tryptophan residue that results from the putative UAG→UGG codon edit (11) is shown in red. The red residue is a result of the genome editing, in which a stop codon changes to W. As a result of the editing, the protein synthesis continues additional 22 residues (which are marked in bold) to yield large sHDAg (L-sHDAg). The bold residues represent the additional residues required to make L-sHDAg. ProtParam (https://web.expasy.org/protparam/) analysis results for S- and L-sHDAg are shown below the putative L-sHDAg sequence. At the bottom of the figure, a prediction of cellular localization for human and snake S-HDAg by WoLF PSORT (18) is presented. The prediction results are shown below the respective sequences: nucl, nuclear localization; extr, extracellular localization; cyto, cytoplasmic localization; mito, mitochondrial localization; cyto_nucl, cytoplasmic and nuclear localization, i.e., dual localization. Download FIG S1, PDF file, 0.1 MB. Copyright © 2019 Hetzel et al. 2019 Hetzel et al. This content is distributed under the terms of the Creative Commons Attribution 4.0 International license.

By aligning the nucleotide sequence of sHDV with those of hHDVs, we were able to locate the genomic and antigenomic ribozymes (Fig. 1B). The ribozymes share several features with the hHDV counterparts, including the active site and surrounding nucleotide residues. The phylogenetic analysis of amino acid sequences of hHDAgs shows that the sHDV and avian HDV-like agents (2) are divergent from the hHDV. sHDAg forms a sister clade to hHDAgs, whereas HDAg of the avian HDV-like agent forms an outgroup for these (Fig. 1C).

**FIG 2:**
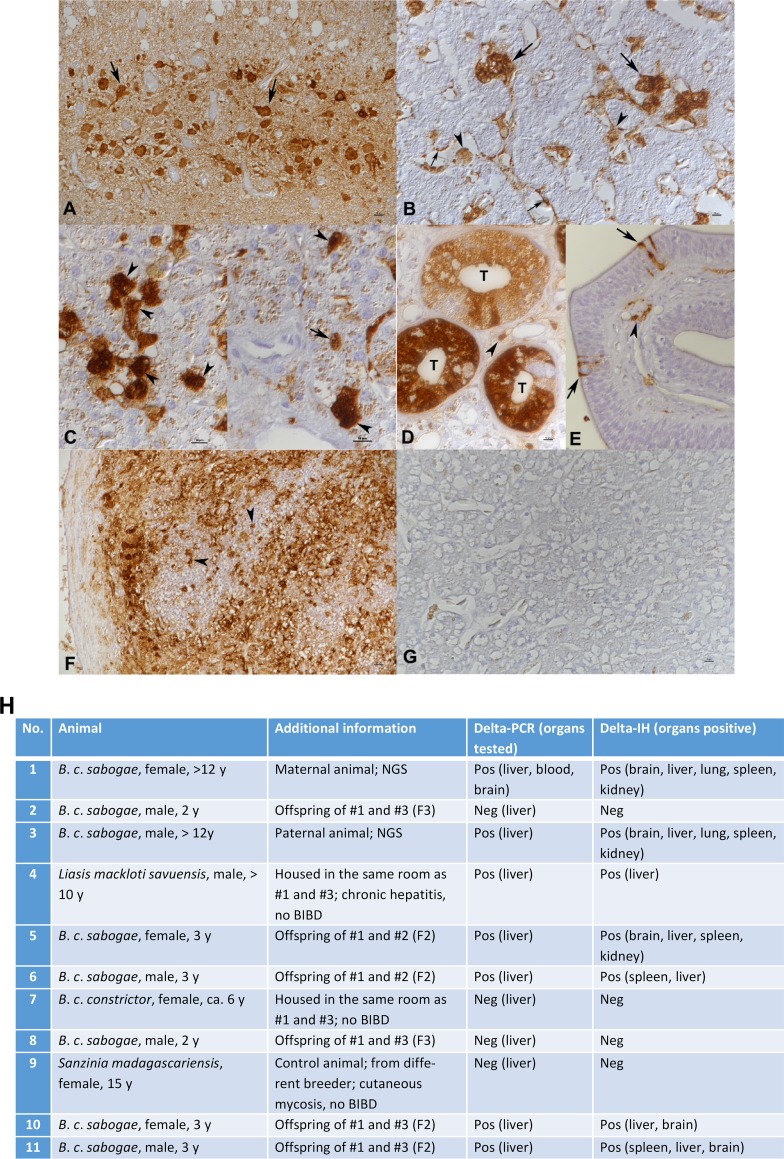
Immunohistology for sHDAg in NGS- and RT-PCR-positive (animals 1 and 3 [A to F]) and negative (animal 7 [G]) animals, and a table of animals included in the study. (A) Brain. Viral antigen is expressed in the nucleus, cytoplasm, and cell processes of numerous neurons. (B) Liver. Individual hepatocytes (large arrows) are strongly positive, and macrophages (arrowheads) and endothelial cells (small arrows) are found to also express viral antigen. (C) Liver. A closer view shows that a substantial proportion of hepatocytes exhibit both cytoplasmic and nuclear (arrowheads) sHDAg expression. On the right, there is also one individual hepatocyte with an exclusively nuclear reaction (arrow). (D) Kidney. In a group of tubules (T), the majority of epithelial cells exhibit variably intense viral antigen expression. Occasional leukocytes in the interstitium (arrowhead) are also positive. (E) Lung. There are several individual positive epithelial cells (arrows); some subepithelial leukocytes are also found to express viral antigen (arrowhead). (F) Spleen. There is extensive viral antigen expression. Positive cells often have the morphology of macrophages (arrowheads). (G) RT-PCR-negative animal (animal 7), liver immunohistology for sHDAg. There is no evidence of sHDAg expression. Horseradish peroxidase method, hematoxylin counterstain. Note that the finely granular brownish staining in some Kupffer cells and hepatocytes in panels C and G is due to bile pigment and/or hemosiderin. (H) Table of animals included in the study.

RT-PCR targeting the nucleotide region 1139 to 1374 was set up using the 5′-GGATTGTCCCTCCAGAGGGTTC-3′ (fwd) and 5′-GCTCGAGGCTACCACCGAAAG-3 (rev) primer pair. We performed conventional RT-PCR as described in reference 10 using RNA extracted from freshly frozen liver samples as the template as described in reference 15. We found the parental animals and four of their seven offspring as well as the water python (animal 4) to be sHDV infected (Fig. 1D and 2H). The latter animal had been housed in the same room as the boa breeding pair for several years, similar to an adult *B. constrictor constrictor* (animal 7) that tested negative for sHDV. A Madagascar tree boa (Sanzinia madagascariensis) without BIBD from a different breeder was equally negative (Fig. 2F). Additionally, we studied 20 blood samples from snakes from a third breeder and found that three of the snakes were positive for sHDV RNA; these results together with Sanger sequencing results of the RT-PCR products are shown in Fig. S2.

10.1128/mBio.00014-19.2 FIG S2 Snake HDV RT-PCR of samples from 20 random snakes from a third breeder. The RT-PCR products were separated on agarose gel, stained using GelRed (Biotium), and visualized under UV light. The strong band in “Pos. CTRL” (positive control) (animal 1 [Fig. 2H]) represents the expected 236-bp RT-PCR product. “Neg. CTRL” (negative control) is nontemplate control. The RT-PCR products from “Snake No. 2,” “Snake No. 4,” and “Snake No. 6” were purified and Sanger sequenced (DNA Sequencing and Genomics, Institute of Biotechnology, University of Helsinki). The Sanger sequencing reads aligned to snake HDV are shown below. Download FIG S2, PDF file, 0.5 MB. Copyright © 2019 Hetzel et al. 2019 Hetzel et al. This content is distributed under the terms of the Creative Commons Attribution 4.0 International license.

To produce an antibody against the sHDAg, we used Champion pET101 Directional TOPO Expression kit (Thermo Scientific) to clone and express the recombinant sHDAg with a C-terminal hexahistidine tag. We designed primers (5′-CACCATGGAAACTCCATCCAAGAAGC-3′ [fwd] and 5′-CGGGAACATTTTGTCACCCCTCAC-3′ [rev]) according to the manufacturer’s instructions to PCR amplify sHDAg ORF from the brain sample used for NGS library preparation. We did the protein expression similarly as described in reference 15 but performed the purification under native conditions using Ni-NTA agarose (Qiagen) according to the manufacturer’s instructions. Rabbit antiserum against the recombinant protein was prepared by BioGenes GmbH as described previously (15). We tested the anti-sHDAg antiserum by Western blotting brain and liver homogenates (prepared as described in reference 16) from the parental animals (animals 1 and 3) and an RT-PCR-negative snake, along with serum samples. At 1:40,000 dilution, using protocols described previously (16, 17), the antiserum detected two bands with estimated molecular weights of 20 kDa and 27 kDa in the liver samples of the infected snakes (Fig. 1E). The bands detected likely represent the S- and L-sHDAg, which have estimated molecular weights of 22.7 kDa and 25.6 kDa, respectively (Fig. S1). The samples of sHDV RT-PCR-negative snakes showed very little background staining. Curiously, only the large ∼27-kDa form of the sHDAg was detected in the brain samples. The serum samples showed huge background, probably due to the presence of snake immunoglobulins. Immunohistology then served to detect sHDAg expression in the formalin-fixed and paraffin-embedded tissues (brain, liver, lung, kidney, and spleen) of the snakes examined, using the anti-sHDAg antiserum. We used the EnVision HRP detection system (Dako) as described previously (15), citrate buffer (pH 6.0 at 98°C, 10 min) for antigen retrieval, and anti-sHDAg serum at 1:10,000 dilution in Dako dilution buffer. Consecutive sections incubated with the preimmune serum instead of the specific primary antibody and tissues from RT-PCR-negative snakes served as negative controls.

In both parental boas, we found sHDAg to be intensely expressed within the cell body and processes of numerous neurons in all brain regions (Fig. 2A), in individual hepatocytes in the liver (Fig. 2B and C), in a proportion of tubular epithelial cells in the kidney (Fig. 2D), in occasional epithelial cells in the lung (Fig. 2E), and in leukocytes (mainly consistent with macrophages) in the spleen (Fig. 2F). In addition to the cytoplasmic reaction, nuclear staining was seen in a proportion of hepatocytes, and some exhibited solely a nuclear reaction (Fig. 2C). All tissues also showed evidence of viral antigen expression in occasional vascular endothelial cells and some leukocytes (Fig. 2). These findings together with the Western blotting results (Fig. 1E) suggest active sHDV replication in various tissues. Of the seven juvenile offspring tested, we found the four RT-PCR-positive animals to also be positive by immunohistology, though mainly with a more limited expression (Fig. 2H). The RT-PCR-positive water python exhibited patchy sHDAg expression in the liver. The three RT-PCR-negative boa offspring and the RT-PCR negative-control animal (animal 7 [Fig. 2H]) were also negative by immunohistology (Fig. 2G). Because we were surprised by the prominent cytoplasmic staining of sHDAg, we used WoLF pSORT (18) to compare the predicted localization of hHDAg versus sHDAg. The prediction results (Fig. S1) corroborate our observations of prominent sHDAg localization in both cytoplasm and nucleus, despite the predicted NLSs. The localization of hHDAg shifts from nuclear to cytoplasmic as a result of HBV S-antigen coexpression (19), and thus, the prominent cytoplasmic localization of sHDAg could be explained by coexpression of glycoproteins of the yet unidentified accompanying enveloped virus.

Herein we provide the first evidence of actively replicating deltavirus in species other than humans. Together with the recent report by Wille et al. (2), our study also suggests that deltaviruses are in fact likely present in several taxa. Evidence of replication in the present study includes the following. (i) Viral RNA is found in variable amounts in tissues and individuals. (ii) The virus is found in some but not all offspring (<100% vertical transmission). (iii) sHDAg expression varies in its extent in positive cells and is not observed in every cell. (iv) L- and S-HDAg are both present in the liver, but only L-sHDAg is found in the brain. Our immunohistological examination shows that the tropism of sHDV is broad and not limited to liver and blood. In fact, the detection of sHDAg in the renal tubular epithelium and lung epithelial cells indicates that the virus can be shed with secretions. We could not associate the infection with cytopathic changes; further studies are required to assess the sHDV-related pathogenesis. The fact that we, like Wille et al. (2), could not detect accompanying hepadnavirus challenges the current understanding of a strict hepadnavirus-deltavirus association. It would seem plausible that the newly found deltaviruses use arenavirus (in the case of snakes) and influenza virus (in the case of birds) coinfection to obtain the lipid envelope to make infectious particles. The ultimate proof of viral replication would require experimental infection of snakes; however, this would ideally use pure components, i.e., sHDV and the so far unknown helper virus. Alternatively, homogenates of different sHDV-infected tissues could be used. Such an approach could provide clues regarding the potential helper virus and could help to reveal the nature of sHDV particles and to identify the potential pathogenicity of sHDV. The present findings open up a multitude of avenues in deltavirus research.
