# Supplementary material for: Identification of a Novel Deltavirus in Boa Constrictors
Source: mBio. 2019 Apr 2;10(2):e00014-19. doi: 10.1128/mBio.00014-19 (PMC6445931; doi:10.1128/mBio.00014-19)
Supplement: FIG S1 [file mBio.00014-19-sf001.pdf]

Amino acid alignment of snake, human and avian HDAg

```
                 1                                                 50
Snake delta Ag   M E T P S K K Q I P T P S R E D I L E Q W V E L G K R K K E L E K E L Q K V T K K K R K L E E Q H G
Human delta Ag   - M S R S E K K K D R G G R E D I L E Q W V S G R K K V E E L E K D L R K V K K K V K K L E E D N P
Avian delta Ag   M E N K E E S Q K K K R G R E E T L Q K W V D D R K R K R E L E E E L E K L R K R I K D R E R K N P

                 51                                                100
Snake delta Ag   F L G N V L G I V R G K E Q K P A A T P Q K K R K A E E S M D V D G G S R L P P K - E I K K R I F T
Human delta Ag   W L G N I K G I I G K K D K D G E G A P P A K R S R T D Q M E V D S G P R K K P - - - - P R G G F T
Avian delta Ag   W L G N L K G M L K - - - Q D S G G G Q E K K K E A A E P M D T G E S P K K K K K K D Y E G L S F T

                 101                                               150
Snake delta Ag   E E E R A E H R R R G Q L E N K K K Q L E G R G K Q L P E E E Q K L L A E L T R K D - - - - - - - -
Human delta Ag   D K E R E D H R R R K T L E N K K K Q L S S G G K R L S R E E E E E L E R L T R E D - - - - - - - -
Avian delta Ag   P E E K Q R H K R K C D L E N K K K Q L N A K G K Q L T S Q E E D E L R N L Q E E D K K R L L K K R

                 151                                               200
Snake delta Ag   E E R K Q R F H Y G G A G E V N P L E G Q S R G A F G G G F V P S T Q G V P E S P F H R T G T G L D
Human delta Ag   E E R K R R V A G P R V G G V N P L E G G S R G A P G G G F V P S L Q G V P E S P F S R T G E G L D
Avian delta Ag   E E R E R R - - - - - S G G V N L F G S A S P S T S G G G N A S S T Q G L - R L P W Q K - - - - - -

                 201         208
Snake delta Ag   V R G D K M F P
Human delta Ag   I R G N Q G F P
Avian delta Ag   - - - - - - - -
```

Amino acid identities between snake, human and avian HDAg

	AYF55701.1 Snake HDAg	AWI66689.1 Human small HDAg	AYC81245.1 Avian HDAg
AYF55701.1 Snake HDAg	100 %		
AWI66689.1 Human small HDAg	55 %	100 %	
AYC81245.1 Avian HDAg	37 %	39 %	100 %

Amino acid sequence of putative L-sHDAg

METPSKKQIPTPSREDILEQWVELG*KRKKELEKELQKVTKKKRKLEEQHGFLGNVLGIVRGKEQKPAAT*
*PQKKRKAEESMDVDGGSRLPPKEIKKRIFTEEERAEHRRRGQLENKKKQLE*GRGKQLPEEEQKLLAELT
RKDEERKQRFHYGGAGEVNPLEGQSRGAFGGGFVPSTQGVPESPFHRTGTGLDVRGDKMFP**WKRNEERR**
SERGEEERSREKPE

S-sHDAg
Number of amino acids: 199
Molecular weight: 22728.8
Theoretical pI: 9.74

L-sHDAg
Number of amino acids: 221
Molecular weight: 25583.9
Theoretical pI: 9.69

WoLF PSORT prediction of human and snake S-HDAg

```
>AAQ09794.1 small delta antigen [Hepatitis delta virus]
MSRSESKRNRDGREGILEQWVNGRKKLEDLEREARKIKKKIKKLEDENPWLGNIKGILGKKDKDGEGAP
PAKRARTDQMEIDSGPGKRPLRGGFSDKERQDHRRRKALENKRKQLAGGKHLSKEEEEELKRLTEEDE
RRERRTAGPSVGGVNPLEGGSRGAPGGGFVPNMLSVPESPFSRTGEGLDVRGNQGFP

WoLFPSORT prediction: nucl: 27, extr: 2, cyto: 2, mito: 1

>AYF55701.1 delta antigen [Snake deltavirus F18-5]
METPSKKQIPTPSREDILEQWVELGKRKKELEKELQKVTKKKRKLEEQHGFLGNVLGIVRGKEQKPAAT
PQKKRKAEESMDVDGGSRLPPKEIKKRIFTEEERAEHRRRGQLENKKKQLEGRGKQLPEEEQKLLAELT
RKDEERKQRFHYGGAGEVNPLEGQSRGAFGGGFVPSTQGVPESPFHRTGTGLDVRGDKMFP

WoLFPSORT prediction: nucl: 25.5, cyto_nucl: 16.5, cyto: 6.5
```
