# Supplementary material for: Identification of a Novel Deltavirus in Boa Constrictors
Source: mBio. 2019 Apr 2;10(2):e00014-19. doi: 10.1128/mBio.00014-19 (PMC6445931; doi:10.1128/mBio.00014-19)

Snake HDV RT-PCR, random snakes from breeder #3

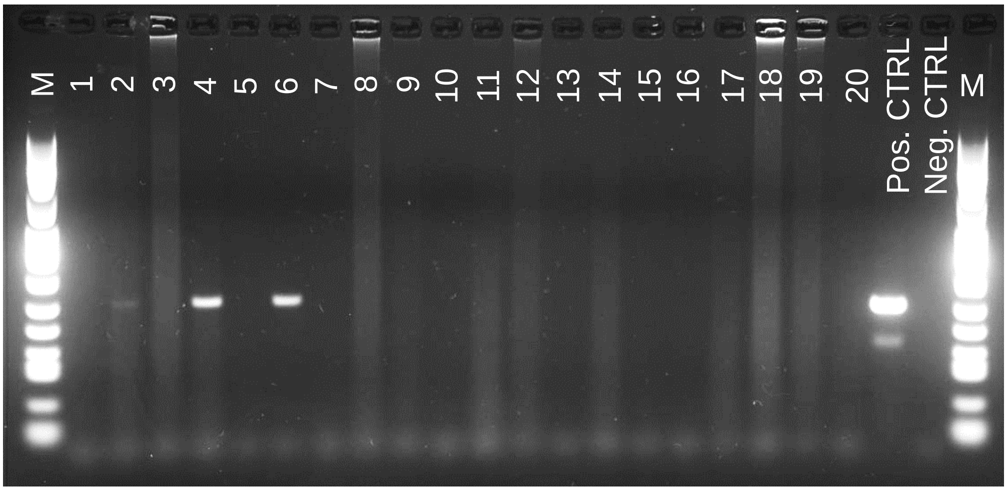

Samples:

1. Snake No. 1
2. Snake No. 2
3. Snake No. 3
4. Snake No. 4
5. Snake No. 5
6. Snake No. 6
7. Snake No. 7
8. Snake No. 8
9. Snake No. 9
10. Snake No. 10
11. Snake No. 11
12. Snake No. 12
13. Snake No. 13
14. Snake No. 14
15. Snake No. 15
16. Snake No. 16
17. Snake No. 17
18. Snake No. 18
19. Snake No. 19
20. Snake No. 20

M = marker
Pos. CTRL, F18-5
Neg. CTRL, non-template control

Sanger sequenced RT-PCR products of snakes No. 2, 4 and 6 aligned to snake HDV database sequence

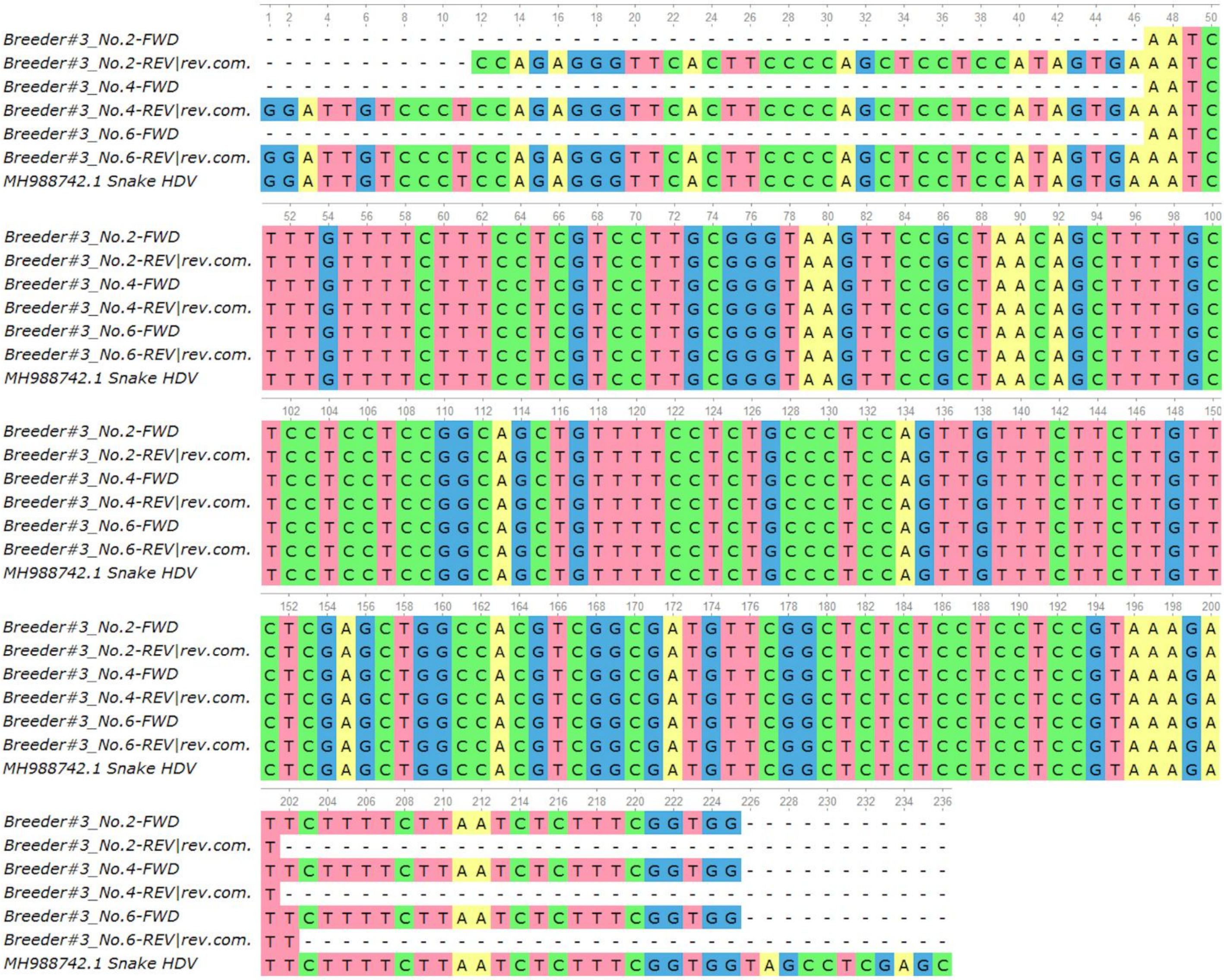

Supplement: FIG S2 [file mBio.00014-19-sf002.pdf]
